# Unveiling Vertebrate Biodiversity in Arid and Semi‐Arid Terrestrial Ecosystems Through eDNA Metabarcoding at Savanna Waterholes

**DOI:** 10.1111/eva.70200

**Published:** 2026-01-29

**Authors:** Tamara Schenekar, Janine Baxter, Irmgard Sedlmayr, Julia Gladitsch, Sibusiso Mahlangu, Monica Mwale

**Affiliations:** ^1^ Department of Biology University of Graz Graz Austria; ^2^ South African National Biodiversity Institute National Zoological Gardens Pretoria South Africa; ^3^ NRF‐South African Institute for Aquatic Biodiversity Makhanda (Grahamstown) South Africa

**Keywords:** 12S rRNA, detection probabilities, eDNA, false positives, mammals, metabarcoding, substrate, terrestrial

## Abstract

Applying environmental DNA (eDNA) metabarcoding to samples from waterholes and their surroundings offers a promising approach for monitoring terrestrial vertebrates in semi‐arid and arid ecosystems, such as the southern African savannas. However, minimal guidance exists on key sampling design parameters for terrestrial ecosystems, which can significantly influence species detection. This study investigated the effects of sampled substrate, sampling season, and metabarcoding primer pair on species richness and taxonomic group detection in terrestrial vertebrates, with a focus on mammals, using eDNA samples from waterholes in Botsalano Game Reserve, South Africa. A total of 725 eDNA samples were collected from 94 sampling events across wet and dry seasons, detecting 95 species (45 birds, 42 mammals, 4 amphibians, 3 reptiles, and 1 fish). Sediment samples provided more reliable detection of abundant taxa, whereas water samples had higher detection frequencies of rare taxa. A mixed sampling approach yielded the highest species richness. Sampling during the wet season yielded higher species richness overall, while more mammal species were detected from dry season sampling. Overlap in species detection between the two metabarcoding primers tested was low (47%). We formulate recommendations for future eDNA metabarcoding study designs in similar systems, including remote sampling logistics and discuss potential sources of false positives in eDNA metabarcoding, including (1) secondary eDNA input, (2) incomplete genetic reference databases, and (3) the low genetic resolution of metabarcoding markers.

## 1 Introduction

The use of environmental DNA (eDNA), defined as extraorganismal DNA shed into the environment and extractable from environmental samples (Taberlet et al. 2012), has proven to be an effective approach in enhancing biodiversity assessments for management applications such as ecosystem health monitoring (Aylagas et al. 2014; Ruppert et al. 2019; Suren et al. 2024; Yang and Zhang 2020), conservation planning (Cristescu and Hebert 2018; Thomsen and Willerslev 2015) or environmental impact assessments (Allan et al. 2023; Laroche et al. 2018). Hereby, eDNA metabarcoding has demonstrated high cost‐ and time‐efficiency in the simultaneous detection of multiple species (Carvalho et al. 2022; Ji et al. 2013; Svenningsen et al. 2022; Valentini et al. 2016), often outperforming or complementing traditional survey methods (Fediajevaite et al. 2021). To date, there has been less focus on the development of this approach in terrestrial ecosystems compared to aquatic environments like rivers, lakes or marine systems (van der Heyde et al. 2022). This is partly due to historical reasons (early eDNA studies on macroorganisms targeting aquatic systems), partly driven by conservation and monitoring needs, but also due to practical factors such as the ease of concentrating eDNA from water through filtration and the relative homogeneity of water, which facilitates uniform sampling. In contrast, terrestrial systems exhibit a far more patchy distribution of eDNA across and within substrates, such as soil, water and plant surfaces, due to their greater heterogeneity on smaller spatial scales (Cowgill et al. 2025; Grosberg et al. 2012). This makes sampling design even more critical for terrestrial studies. To date, the most frequently sampled substrate for terrestrial systems is soil, followed by stomach contents/fecal material and water (Cowgill et al. 2025; van der Heyde et al. 2022). However, the optimal sampling substrate may vary depending on the target taxa (van der Heyde et al. 2020) and, although rarely employed, combining multiple substrates often increases detected species richness (Cowgill et al. 2025; van der Heyde et al. 2022). The temporal aspect of sampling is equally crucial and has also been shown to influence eDNA‐based species detection (Buxton et al. 2018; Djurhuus et al. 2020; Johnson et al. 2021). This is either due to different physical conditions of the environment across seasons, affecting eDNA persistence, or different activity patterns of the target organisms, affecting eDNA deposition (Buxton et al. 2017; Salter 2018). However, only a small number of eDNA‐based studies for environmental monitoring focus on the temporal variation in detected biodiversity (Mathieu et al. 2020). In terrestrial environments, the majority of eDNA‐based studies (targeting monitoring of ecological restoration) use only a single sampling period (van der Heyde et al. 2022). The choice of the metabarcoding primer pair is a third key parameter in any eDNA metabarcoding analysis. They define the taxonomic target group of the eDNA metabarcoding assay and have been shown to affect species detection, even when targeting similar taxonomic groups (Schenekar et al. 2020; Zhang et al. 2020). Multiple universal primer pairs are increasingly used to maximize species detection, despite higher laboratory costs (Elbrecht et al. 2017; van der Heyde et al. 2022).

The biomes of the African continent still remain among the least explored systems concerning eDNA dynamics and application (Belle et al. 2019; Cowgill et al. 2025; Schenekar 2023; von der Heyden 2022). However, their exceptional biodiversity and persisting anthropogenic threats (Archer et al. 2021; Myers et al. 2000) demand efficient and cost‐effective monitoring techniques to inform conservation efforts and guide management policies. In semi‐arid to arid systems like savannas, waterholes (both natural and artificial) serve as essential aggregation points for wildlife seeking scarce water resources, making these critical locations for monitoring and management efforts (Redfern et al. 2005; Sutherland et al. 2018). Few studies have started to explore the applicability of waterhole‐borne eDNA to monitor pathogen (Alfano et al. 2021; Farrell et al. 2019) or mammal (Farrell et al. 2022; Li et al. 2023; Mas‐Carrió et al. 2022) biodiversity in Africa. Despite these studies revealing the high potential of waterhole‐borne eDNA to monitor terrestrial wildlife, a systematic evaluation of the variability and reliability of eDNA metabarcoding while testing for multiple workflows is lacking.

This study examines how various sampling design and workflow parameters affect eDNA metabarcoding results for monitoring terrestrial vertebrates in savanna systems using samples collected from waterholes and their immediate surroundings. This was conducted through two experimental setups. The first experiment aimed at understanding the primary factors such as substrate, season, and primer affecting the detection of terrestrial vertebrate species. The second experiment evaluated the potential use of soil samples from wildlife trails near waterholes as an alternative to direct sampling of water or sediment from waterholes.

## 2 Materials and Methods

### 2.1 Study Site

**FIGURE 1:**
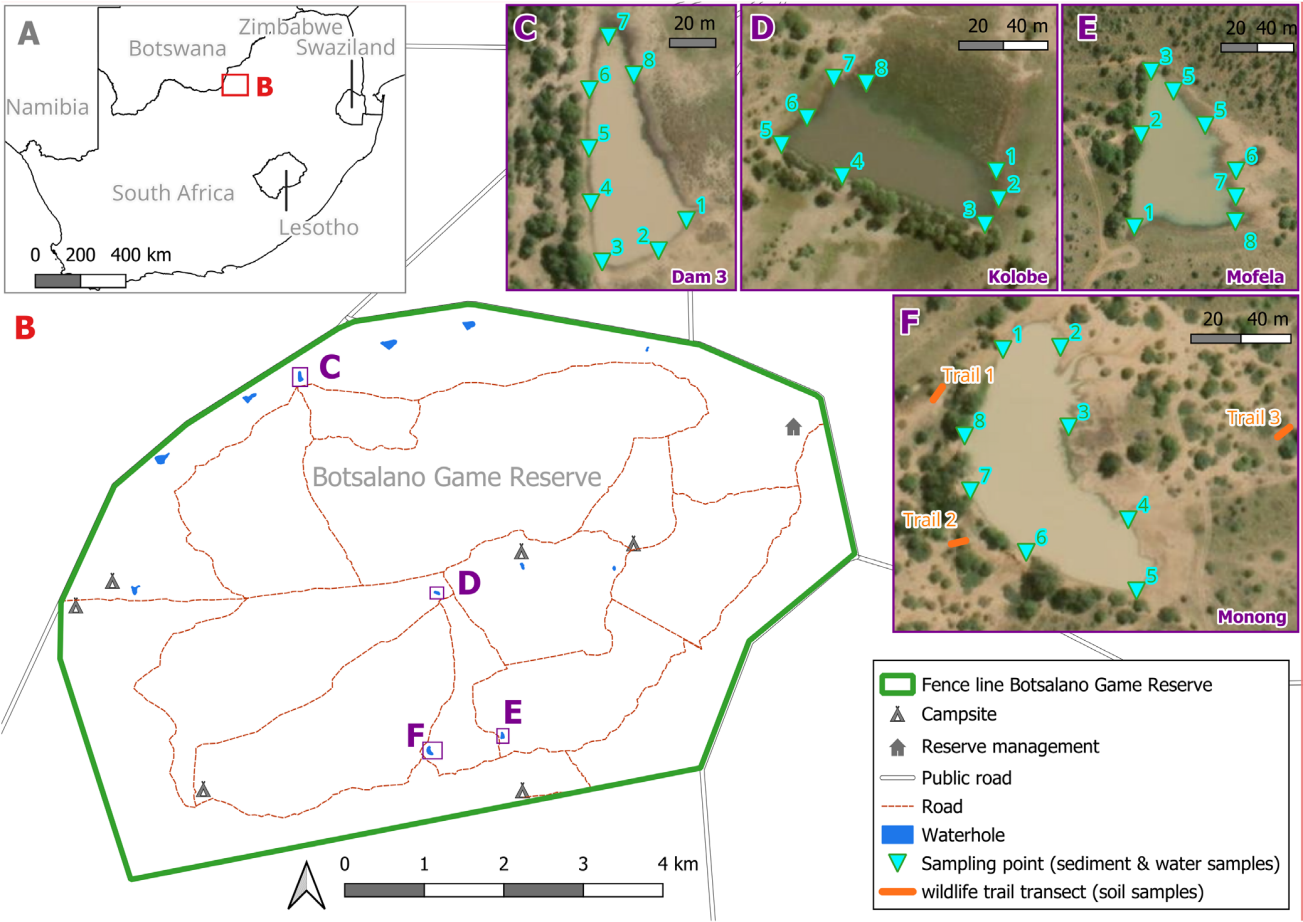
Location of Botsalano Game Reserve (A) as well as the sampled waterholes and wildlife trails within the reserve (B). Approximate sampling points at each of the waterholes are shown in (C–F). Satellite images: ESRI (2024).

The study was conducted in Botsalano Game Reserve, a 6000‐ha game reserve situated in North West Province, South Africa (Figure 1). The prevailing climate is semi‐arid with hot, rainy summers (October to April) and cold, dry winters (May to September). During our sampling in two seasons in 2023 (see below) precipitation differed substantially between sampling seasons; average temperatures were relatively equal (Material S1, Figure S1).

Botsalano Game Reserve lies within the savanna biome in the highveld of South Africa, encompassing umbrella thorn ( *Vachellia tortilis* ) savanna and woodland as well as black thorn scrub (*Senegalia mellifera*; Morris 2022). The topology is characterized by a relatively flat to rolling terrain and low‐relief hills, ranging in elevation approximately between 1300 and 1440 m above sea level. Within the reserve, dams have been constructed along ephemeral streams to hold water during the dry season, resulting in semi‐natural waterholes. The ephemeral streams fall dry soon after the main rainfalls (November–February), so that all waterholes represent independent water bodies. The reserve does not conduct regular game counts for species inventory, although Morris (2022) lists more than 100 bird species and over 20 larger‐bodied mammals (Material S1, Table S1).

Three waterholes in the reserve were selected during each season based on accessibility, spatial distribution, and anticipated water levels during the wet and dry seasons. Although the initial sampling design intended to use the same waterholes across both seasons, one waterhole (Dam 3) dried up at the start of the dry season and was replaced with an alternative waterhole (Monong) during sampling. Consequently, the waterholes sampled during the wet season were Kolobe, Mofela, and Dam 3, while Kolobe, Mofela, and Monong were done during the dry season (Figure 1).

### 2.2 Sample Collection and Processing in the Field

Sampling was conducted during two seasons in 2023: at the end of the wet season (March 10th to April 23rd, 2023) and at the end of the dry season (September 9 to October 23, 2023). Each waterhole was sampled eight times per sampling season, with 6‐day intervals between sampling days. Due to low water levels during the dry sampling season, the number of sampling events varied across waterholes for this season (Kolobe—water 2×, sediment 5×; Mofela—water 5×, sediment 8×; Monong—water and sediment 7×). Wildlife trails were sampled in the dry season only, at four sampling days, also using 6‐day intervals. A ‘sampling event’ hereafter refers to the collection of a specific substrate (water, sediment, or soil) from a specific location (e.g., Waterhole ‘Mofela’ or ‘Trail 1’; see below) on a given day of sampling. For each sampling day at a waterhole, eight replicate samples of both water and sediment were collected. For water sampling, a DNA‐free 500 mL Nalgene bottle mounted to a 2 m PVC tube was utilized to scoop up surface water approximately 1.5 m away from the shoreline (Material S1, Figure S2a). For sediment sampling, approximately 3 mL of sediments, still submerged in water, were collected directly at the shoreline and transferred into a 15 mL tube using a DNA‐free spatula (Figure S2b). During each sampling event at a waterhole, pH, conductivity, and dissolved oxygen levels of the water body were measured.

For wildlife trail soil sampling, three trails around Monong waterhole, located within a 60 m radius, were selected for the placement of line transects for defining sampling points (Figure 1F). Along each trail, a 5 m transect was established using a rope marked at 1 m intervals, creating six evenly spaced sampling points per transect from which six soil samples were collected per sampling event. Trails were sampled at 6‐day intervals (all three trails on the same day) over a total of four sampling events. At each sampling point, approximately 3 mL of surface soil was collected from a 3 × 3 cm area using a DNA‐free spatula and placed in a 15 mL tube (Material S1, Figure S3).

All samples were kept refrigerated until filtering (water samples) or preservative buffer was added (sediment and soil samples) within a maximum of 5 h after sampling.

Water samples were filtered using a 250 mL Nalgene filter holder mounted onto a 1 L vacuum flask, powered by a peristaltic pump (MasterFlex peristaltic vacuum pump LS 600R) via a MasterFlex L/S 15 silicone tube and pumped at a maximum speed of 700 mL/min. Filtering was conducted until either the entire volume of 500 mL was processed or after a maximum filtration time of 10 min per filter. In the wet season, all samples were first prefiltered through a 20 μm Nylon filter, followed by filtration through a 3 μm MCE filter. If less than 250 mL was filtered through the 3 μm MCE filter, the remaining sample was then filtered through an 8 μm MCE filter, and this filter was used for DNA extraction and metabarcoding. In the dry season, which involved higher water turbidity, increased suspended particle load and greater filter clogging, the 20 μm Nylon filter was used directly for DNA extraction and metabarcoding. Filters were transferred to 5 mL tubes containing 2.5 mL Longmire's solution with added sodium azide (Longmire et al. 1997). For sediment and soil samples, 6 mL of Longmire's solution was added to each sample. All samples were stored at 8°C in the field until transport to the genetic laboratory (up to 7 weeks) where they were stored at −20°C until DNA extraction. All sampling and filtering equipment (e.g., sampling bottles, PVC sampling tube, spatulas, filter holder, forceps, etc.) were cleaned between sampling events and waterholes by soaking in 30% bleach for 30 min followed by soaking in distilled water for 30 min. On each day, a field negative control was processed by filtering 150 mL of distilled water before processing the first sample of that day.

### 2.3 DNA Extraction, Metabarcoding Library Preparation and Sequencing

DNA extractions and setup of PCRs were conducted in a dedicated low‐template DNA laboratory under two separate laminar‐flows for DNA extractions and PCR setup. One extraction blank was processed with every DNA extraction batch (up to 11 samples per batch) and laminar‐flow work benches were irradiated with UV light for 30 min between work sessions. DNA extractions were carried out with the DNeasy PowerSoil Pro Kit (Qiagen) using the protocol of Schenekar et al. (2024) in a final elution volume of 100 μL (extraction protocol: Material S2). For metabarcoding library preparation, two primer pairs were utilized targeting two different fragments of the mitochondrial 12S rRNA fragment: 1. The MiMammal primer set designed to amplify mammalian sequences and 2. The 12SV5 primer designed to identify vertebrate sequences (Table 1). Both primer pairs were fitted with eight‐base‐pair (8‐bp) indices on the 5′ ends of both the forward and reverse primers, respectively, to enable sample demultiplexing using unique dual indices (UDIs). A total of four PCR (polymerase chain reaction) replicates were analysed per sample for amplification of both markers and then pooled. Each PCR consisted of 1.5 μL template DNA, 0.25 μL Q5 High‐Fidelity Polymerase (2000 U/mL, New England Biolabs), 2.5 μL Q5 Reaction Buffer, 0.2 mM dNTPs, 0.3 μM of forward and reverse primer, respectively, and nuclease‐free water up to a final reaction volume of 12.5 μL. Cycling conditions were as follows: Initial denaturation at 98°C for 30 s, followed by 40 cycles of denaturation at 98°C for 20 s, annealing at 64°C (MiMammal) or 57°C (12SV5) for 30 s, extension at 72°C for 60 s and a final extension at 72°C for 7 min. Due to co‐amplification of non‐target fragments from algae and bacteria in the sediment and water samples (taxa confirmed by Sanger Sequencing of individual selected samples), resulting in double bands, all PCR products were excised from a 2% agarose gel and subsequently cleaned using the Monarch Spin DNA Gel Extraction Kit (New England Biolabs). Each PCR batch encompassed the amplicons of 48 samples per marker and included two controls: a PCR positive control/mock community and a PCR negative control (HPLC‐purified water). The mock community contained the DNA extracts (7 ng/μL) of six non‐native mammals, namely Eurasian beaver ( *Castor fiber* ), European hedgehog ( *Erinaceus europaeus* ), raccoon ( *Procyon lotor* ), brown bear ( *Ursus arctos* ), Eurasian fish otter ( *Lutra lutra* ) and roe deer ( *Capreolus capreolus* ). Field and extraction negative controls were processed in the same manner as field samples. A total of 192 amplicons (96 samples, two markers each) were pooled per library and sent to Novogene for final library preparation (using the NEBNext Ultra II DNA PCR‐free Library Prep Kit for Illumina, New England Biolabs) for Illumina adapter incorporation and Illumina sequencing. Libraries were sequenced on an Illumina NovaSeq X in 150 bp paired‐end read mode with a targeted output of 80 million reads per library (417,000 reads per sample and marker).

**TABLE 1:** The primer pairs utilized for DNA metabarcoding.

Primer pair	Primer sequences	Amplicon length	References
12SV5	Forward: 5′‐XXXXXXXXYAGAACAGGCTCCTCTAG‐3′	98 bp	Riaz et al. (2011)
	Reverse: 5′‐XXXXXXXXTTAGATACCCCACTATGC‐3′		
MiMammal	Forward: 5′‐XXXXXXXXGGRYTGGTHAATTTCGTGCCAGC‐3′	171 bp	Ushio et al. (2017)
	Reverse: 5′‐XXXXXXXXCATAGTGRGGTATCTAATCYCAGTTTG‐3′		

*Note:* Given are the primer pair name, the sequences of forward and reverse primer, respectively, the approximate target amplicon length and the original reference of the primer pair. XXXXXXXX indicate 8‐bp index sequences that were used for UDI sample demultiplexing.

### 2.4 Bioinformatic Analysis and Taxonomic Classification of ASVs

Raw reads were quality‐checked with FastQC (Andrews 2010). Quality filtering was conducted with the *fastx_filter* function of *vsearch* v2.15.1 (Rognes et al. 2016) with the maximum error rate set to fastq_maxee = 1. Paired‐end reads were merged using the *fastq_mergepairs* function, using the default settings and allowing for merging of staggered reads. Samples were demultiplexed by indices (1st level) and by primer pair (2nd level), allowing for one error in the index and allowing for two errors in the primer sequences using *cutadapt* 4.1 (Martin 2011). *Cutadapt* was subsequently also used for length filtering. Hereby, only reads with a minimum length of 85/155 bp and a maximum length of 115/190 bp were kept for 12SV5 and MiMammal, respectively. Unique amplicon sequence variants (ASVs) were created from the entire dataset using the *derep_fulllength* function, and denoising was conducted by only retaining ASVs that had a minimum read count of five reads and a minimum alpha of 2 using the *unoise_alpha* algorithm of *vsearch*. Chimera sequences were removed from the ASV dataset using the *uchime3_denovo* function of *vsearch*. Quality‐filtered reads were mapped to ASVs using the *usearch_global* function with a threshold of 99% similarity. Taxonomic assignment was done using the srRNA_SINTAX database of MIDORI2 (v. GB261; Leray et al. 2022) via the *sintax* algorithm of *vsearch* and a sintax_cutoff of 0.9. ASVs that were assigned to the same species were amalgamated to that species for further statistical analysis.

A list of previously documented species in South Africa was retrieved querying the GBIF database (GBIF.org 2025) using the following settings: Country: South Africa, Year: 1950 and later; Basis of record: Observation and Human Observation. For detected species that did not have any occurrence records (hereafter called “non‐documented species”), a species list of congeneric species that do have occurrence records based on these criteria was compiled. Additionally, a species list with the same criteria was compiled for all classifications that could only be resolved to the genus level. Both species lists were checked against the MIDORI2 database, using a custom bash script, to check which species lacked 12SrRNA records. For taxonomic assignment, if the genus or the non‐documented species could be matched to a previously documented species, i.e., only one congeneric species occurring in South Africa, it was assigned to that species. If the non‐native species or genus could not be unambiguously assigned to one native species, the taxon and corresponding ASVs were excluded from the analysis, with the reason for exclusion listed in Table S2. Furthermore, any detection of domestic animals, humans, or that likely stem from anthropogenic input through food or contamination (e.g., *Thunnus albacares, Salmo salar*) was discarded. The only exception was the detection on *Canis lupus* , being re‐classified as *Canis mesomelas* /*Lupulella mesomelas* (black‐backed jackal) as this was an abundant native species in the reserve that has only one genetic reference sequence in MIDORI2 (Detailed justification for this taxonomic re‐assignment: Material S3).

### 2.5 Statistical Analysis

Statistical analyses were performed separately for each primer pair dataset (12SV5 and MiMammal), and as a combined data set including detections from either primer pair. Species richness (number of detected species) was calculated across the full dataset and separately by location (e.g., Kolobe, Monong, Trail1), substrate (water, sediment, soil), season (dry/wet), and primers. Species richness was also assessed for all taxa combined and by taxonomic class. Euler diagrams of shared/exclusive species were created using *eulerr* (Larsson 2024) in *R 4.4.2* (R Core Team 2023) with *RStudio* (Posit Team 2024). For each detected species, the number of positive samples per sampling event was recorded (combined dataset only). For further analyses, species detections from all samples per sampling event were pooled (eight samples for water/sediment, six for soil). Kruskal–Wallis tests assessed richness differences by location, substrate, season, and primer. Nonmetric Multidimensional Scaling (NMDS) plots were created using *metaMDS* of *vegan* (Oksanen et al. 2025) using Jaccard similarity distances. Permutational Multivariate Analysis of Variance (PERMANOVA, 999 permutations; Bray–Curtis dissimilarity distances) was conducted using *adonis2* of *vegan* to assess the effects of location, substrate, season, primer and days since first sampling day on detected species composition. Species accumulation curves were generated with *specaccum* (*vegan*). Species‐specific detection frequency was calculated as the proportion of sampling events detecting a species (per substrate and overall). A Wilcoxon Signed‐Rank Test compared detection frequencies between birds and mammals, and between sediment and water, the latter for all taxa combined as well as for birds/mammals separately. Species were categorized as “rare” (overall detection frequency < 0.1) or “abundant” (≥ 0.1), and detection frequencies were compared between these two groups. A hypothetical mixed‐substrate dataset was created as follows: (1) For all sampling days where eight water samples and eight sediment samples were collected (*N* = 38), we calculated mean species richness per sampling event as well as overall species richness for (a) sediment (all eight sediment samples), (b) water (all eight water samples), and (c) mixed‐substrate samples (four randomly chosen sediment samples and four randomly chosen water samples). Kruskal–Wallis tests evaluated whether mean species richness differed among these sampling approaches. Finally, we tested whether filtered water volume per sampling event had an effect on detected number of species per sampling event (water samples only) via negative binomial regression. Data handling and data visualization used *dplyr* (Wickham et al. 2023), *readr* (Wickham, Hester, and Bryan 2024), *tidyr* (Wickham, Vaughan, and Girlich 2024), *ggplot2* (Wickham 2016), *gridExtra* (Auguie 2017), *cowplot* (Wilke 2024) and *RColorBrewer* (Neuwirth 2022).

## 3 Results

A total of 725 field samples (301 water, 352 sediment and 72 soil samples) were collected in 94 sampling events, along with 38 field negative controls across both sampling seasons (Table 2, Table S3). Overall water temperature was lower and conductivity was considerably higher in dry season. The mean filtered volume of water samples collected in the dry season was larger than those from the wet season despite higher turbidity and conductivity due to the larger pore size of the filters used in this season.

**TABLE 2:** Overview of field sample numbers (field samples) for each waterhole and wildlife trail, separated by season. For water samples, the filtered volume (mean ± SD) is given. Additionally, the mean values of the physicochemical parameters for the three water holes during the two sampling seasons are given.

Season	Waterhole	Field samples	Filtered volume (water samples only) [ml]	Physicochemical parameters of water			
				*T* (°C)	pH	Cond. (μS/cm)	O_2_ (mg/L)
Dry	Kolobe	53	486.9 (±45.3)	14.9	7.4	278.7	6.9
	Mofela	104	473.1 (±81.3)	14.5	7.3	437.5	5.7
	Monong	112	442.9 (±119.6)	16.6	7.8	198.8	6.9
	Trail 1	24	—				
	Trail 2	24	—				
	Trail 3	24	—				
	Overall	341	459.2 (±101.6)				
Wet	Dam 3	128	107.5 (±57.0)	16.6	7.2	104.5	5.5
	Kolobe	128	354.2 (±108.7)	17.7	7.1	68.0	5.6
	Mofela	128	356.0 (±129.5)	17.9	8.0	229.1	5.9
	overall	384	272.6 (±155.7)				

A total of 878 samples (725 field samples, 38 field negative controls, 77 extraction blanks, 19 PCR positive controls, 19 PCR negative controls) were sequenced. Total raw data output was 759,743,937 reads, with 499,499,743 being retained after read quality filtering, pair merging, demultiplexing and length filtering. Dereplication identified 402,263 initial ASVs for 12SV5 and 724,237 initial ASVs for MiMammal. Of these, 2566 and 1683 were kept after denoising and chimera removal for 12SV5 and MiMammal, respectively. Of the retained ASVs, 354/492 could be assigned to species level and 104/178 genus level. A total of 410,190,196 reads (212,417,004 for 12SV5 and 197,773,192 for MiMammal) were assigned to an ASV at species or genus level and were used in the final dataset. Among these, field samples had on average 273,504 reads assigned for the 12SV5 dataset and 250,875 reads assigned for the MiMammal dataset, with the field and laboratory blanks having on average less than 10% of those read numbers (except for field blanks for MiMammal at 16%, Tables S4 and S5).

**FIGURE 2:**
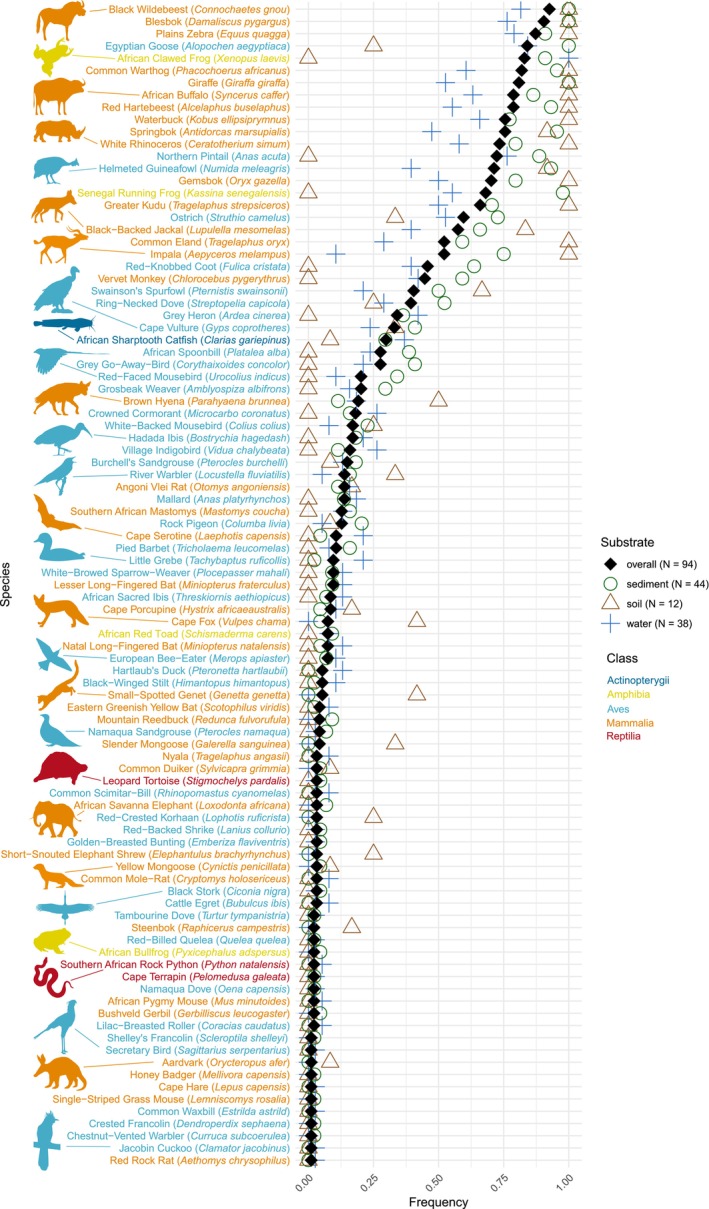
All 95 species detected by eDNA metabarcoding of sediment, soil and water samples, sorted by their overall detection frequencies in the 94 sampling events (black diamonds). Colored symbols (green circles, brown triangles and blue crosses) indicate the detection frequencies across sampling events in the respective substrate of each species.

**FIGURE 3:**
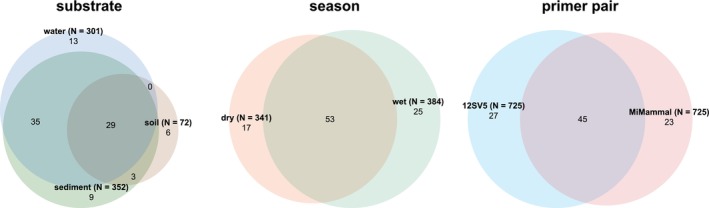
Euler diagrams showing the number of species detected across datasets for different substrates, seasons, and primer pairs. Bold labels indicate the dataset names as well as the number of samples in brackets. Regular font numbers represent the number of species unique to a dataset or shared between datasets in the respective overlaps. Diagrams for substrate and season show the dataset over both primer pairs combined.

Initially, sequences were identified as belonging to 130 species and to 44 genera in the entire sequencing dataset. After taxonomic reassignments based on GBIF occurrences, 95 species were retained in the final dataset (Table S2, Figure 2). The 12SV5 dataset detected 72 species while 68 species were detected with the MiMammal dataset (Table 3). The highest species richness was observed at Mofela Dam, which also had the largest number of samples and sampling events. Sediment and water samples revealed nearly equal species richness, with 76 and 77 species detected, respectively, while only 38 species were detected from soil samples. Water samples had the highest number of exclusive species (Table 3), while soil had the lowest. Overall, more species were detected during the wet season (*n* = 78) compared to the dry season (*n* = 70). A total of 29 species (31%) were detected in all three substrates, 53 species (56%) were detected in both seasons and 45 species (47%) were detected by both primer pairs (Figure 3). Kruskal‐Wallis tests showed significant differences in the number of detected species among locations (*H* = 24.8, *p* < 0.001), substrates (*H* = 19.6, *p* < 0.001) and between primer pair datasets (*H* = 11.0, *p* < 0.001) but not between seasons (*H* = 0.4, *p* = 0.537).

**TABLE 3:** Number of species detected across different locations, substrate and seasons. For each dataset, the number of samples (samples), sampling events (sampling events), total number of detected species (species richness), and number of species that were detected only in the respective dataset (exclusive species) are shown. Data is given for 12SV5 and MiMammal datasets, as well as the combined dataset.

	Samples	Sampling events	12SV5		MiMammal		Combined	
			Species richness	Exclusive species	Species richness	Exclusive species	Species richness	Exclusive species
*Grouped by location*								
Dam 3	128	16	24	3	24	0	24	0
Kolobe	181	23	49	1	45	2	64	2
Mofela	232	29	67	15	54	8	81	17
Monong	112	14	36	3	36	4	51	3
Trail 1	24	4	22	0	25	0	31	0
Trail 2	24	4	24	1	25	2	32	1
Trail 3	24	4	14	0	17	0	19	0
*Grouped by substrate*								
Sediment	352	44	56	8	54	5	76	9
Soil	72	12	29	4	29	5	38	6
Water	301	38	58	12	56	9	77	13
*Grouped by substrate*								
Dry	341	46	51	45	56	15	70	17
Wet	384	48	8	21	53	12	78	25
*Total*	725	94	72	26	68	23	95	*NA*

**FIGURE 4:**
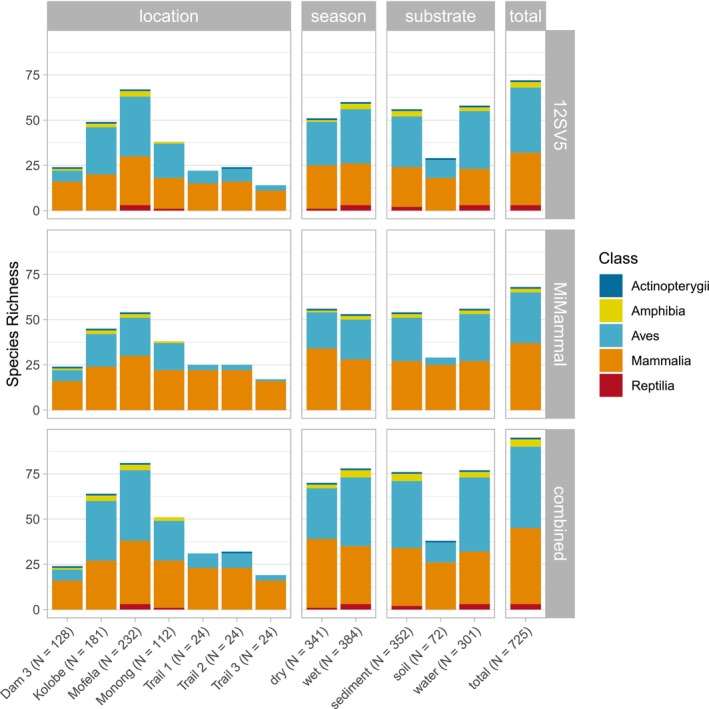
Bar plots showing species richness of each dataset, grouped by location, season, substrate, and overall (total). Results are presented separately for the two primer pairs (12SV5 and MiMammal) and the combined dataset. Colored sections within each bar represent vertebrate classes. Labels on the x‐axis give sample sizes in brackets.

Overall, birds were the most species‐rich class (45 species), followed by mammals (42 species). The 12SV5 primer pair identified species from Actinopterygii, Amphibia, Aves, Mammalia, and Reptilia. Notably, the MiMammal primer pair detected species from all these classes, except Reptilia (Figure 4, Table S6) and identified more mammal species (*n* = 37) than the 12SV5 primer pair (*n* = 29). Seasonal differences in taxonomic composition were observed, with mammal species comprising 40.3% of the total species detected during the wet season and 54.2% during the dry season, while the proportions of bird and reptile species were higher in the wet season (Figure 4). Soil samples had reduced overall species richness, primarily due to fewer bird species (*n* = 11), compared to water (*n* = 41) and sediment (*n* = 37) samples. No amphibian and reptile species were detected from the soil.

The most frequent number of positive samples per species detection in a sampling event was 1 across all substrates, reflecting eDNA's patchy distribution. Sediment and water showed right‐skewed distributions (median: 2), while soil had a more uniform distribution (median: 4; Material S1, Figure S4).

**FIGURE 5:**
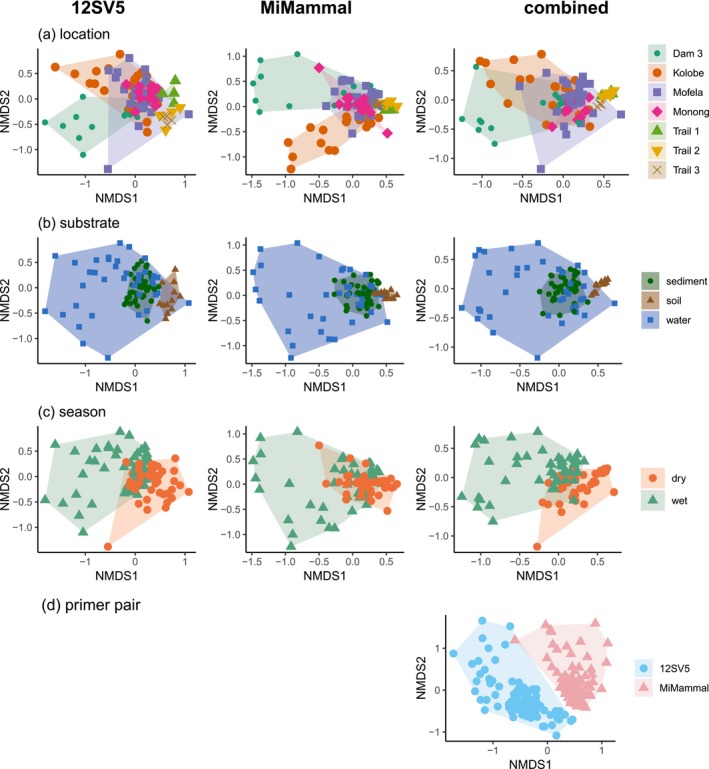
Nonmetric multidimensional scaling (NMDS) plots presenting the differences in species composition among sampled locations (a), substrates (b), between seasons (c), and primer pairs (d). NMDS was performed for the 12SV5 dataset only (column 1), the MiMammal dataset only (column 2), and over both datasets combined (column 3).

NMDS plots of the combined dataset show partial overlap among locations (Figure 5) and water samples covered a much larger area in ordination space compared to sediment and soil samples, revealing a higher dissimilarity of detected species among water samples than among sediment or soil samples. The convex hulls for the dry and wet seasons showed partial overlap while occupied areas of the two primer pairs were essentially non‐overlapping. PERMANOVA analysis showed a significant effect of primer pair (*F* = 110.1, *p* < 0.001), substrate (*F* = 25.0, *p* < 0.001), location (*F* = 8.3, *p* < 0.001), and season (*F* = 7.6, *p* < 0.001) on species composition, while time since first sampling day had no significant effect (*F* = 1.4, *p* = 0.214).

**FIGURE 6:**
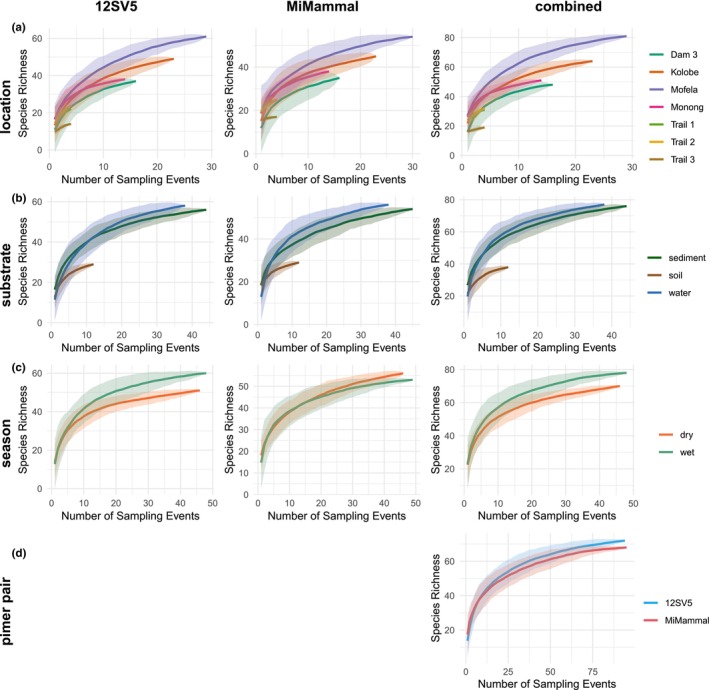
Species accumulation curves showing the number of species detected as a function of the number of sampling events conducted, comparing the sampled locations (a), substrates (b), sampling seasons (c), and primer pairs (d) of this study. Solid lines represent the mean values of species richness, with shaded areas indicating the 95% confidence intervals.

While the slope of most species accumulation curves significantly flattened between five and 10 sampling events, none of the curves fully levelled off, indicating that new and rarer taxa continued to be detected even after 80 or more sampling events (Figure 6). Notably, sediment samples detected more species within the first five sampling events, while water samples identified more taxa overall with increased sampling. Both primer pairs detected similar numbers of species after up to 10 sampling events. However, the 12SV5 primer detected more species as sampling efforts increased.

Detection frequencies of species ranged from 0.01 to 0.93 (Figure 2, Table S7) and did not significantly differ between birds and mammals (*W* = 828, *p* = 0.321) but sediment samples resulted in overall higher detection frequencies than water samples (*V* = 2779, *p* = 0.001). Sediment samples also yielded higher detection frequencies for abundant species (*V* = 944, *p* < 0.001), although this pattern was reversed for rare species (*V* = 325, *p* = 0.048). Furthermore, sediment samples yielded significantly higher detection frequencies than water samples for mammals (*V* = 551, *p* = 0.003), whereas there was no difference in detection frequencies between sediment and water samples for birds (*V* = 638, *p* = 0.096).

**FIGURE 7:**
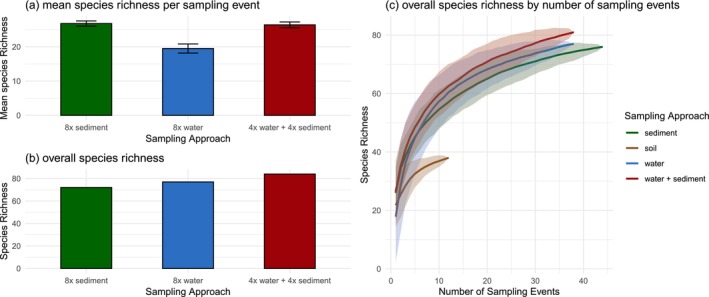
Comparison of the mixed sampling approach (four water samples and four sediment samples per sampling event) to the two single‐substrate sampling approaches (eight sediment samples or eight water samples per sampling event). (a) Mean species richness per sampling event with error bars showing standard deviation. (b) Overall species richness over all sampling events (38 for water and mixed, 44 for sediment). (c) Species accumulation curves for the three sampling approaches. Shaded areas show 95% confidence intervals.

The mixed sampling approach resulted in a mean species richness per sampling event similar to sediment‐only sampling (26.4 ± 0.9 and 26.8 ± 0.8, respectively, Wilcoxon rank sum test, *p* > 0.05), but was higher than water‐only sampling (19.5 ± 1.3, both *p* < 0.001, Figure 7a, Kruskal–Wallis rank sum test; *χ* ^2^ = 22.5, Wilcoxon rank sum tests, both *p* < 0.001). Overall species richness was higher for the mixed sampling approach (84 species) compared to either single‐substrate approach (72 species for sediment and 77 for water, Figure 7b). The mixed approach consistently yielded higher species richness estimates independent of sampling event numbers, although 95% confidence intervals showed substantial overlap with those of the sediment and water‐only approaches (Figure 7c).

Concerning water samples only, we observed a significant correlation between filtered water volumes and detected species per sampling event (*z* = 2.78, *p* = 0.005).

## 4 Discussion

### 4.1 Overall Species Richness, Potential False Positives and False Negatives

The 95 species detected in this study included 19 of the 20 mammal species documented for the game reserve by Morris (2022). The missing mammal was the common reedbuck ( *Redunca arundinum* ), one of the rarest antelopes in the reserve. Moreover, 23 additional mammals not listed by Morris (2022) were also detected. Of the 100 most common birds recorded for pentad 2530_2540 between 2007 and 2022 (African Bird Atlas Project 2022), which encompasses most of Botsalano Game Reserve, eDNA metabarcoding detected 25 species, along with 20 additional species not ranked among the top 100. The initial taxonomic assignment revealed a relatively high number of vertebrate species overall without occurrence records in South Africa (39). A small portion of these detections could be attributed to anthropogenic activity, but the majority of the remaining cases were very likely due to taxonomic misclassifications, as congeneric documented species frequently lacked reference DNA sequences for the target fragments published in the MIDORI database (Table S2). However, even with the stringent filtering steps we applied, five species are still likely to represent false positives. First, neither the African elephant ( *Loxodonta africana* ) nor the nyala ( *Tragelaphus angasii* ) have been introduced to the reserve, and the habitat around Botsalano Game Reserve does not meet the species' requirements, making their presence both within and around the reserve highly unlikely. Neither DNA has ever been processed in any facility involved in the laboratory workflow, excluding contamination during sample processing. No close relatives of the African elephant (Asian elephant, manatees, and dugongs) live nearby the sampling area, rendering taxonomic misclassification for this species unlikely. For nyala, two congeneric species occur in the reserve, namely the eland (*Tragelaphus oryx*) and the kudu ( *Tragelaphus strepsiceros* ), but the respective ASVs assigned to the nyala clearly clustered to the two available genetic reference sequences of the nyala as opposed to kudu and eland (data not shown). We hypothesize that the detection of both elephant and nyala DNA originates from secondary DNA input via birds, most likely vultures, which may have acquired the DNA from carcasses or feces in one of the neighboring reserves harboring these species (e.g., Madikwe Game Reserve—105 km away or Pilanesberg National Park—130 km away). All samples in which nyala or elephant DNA was detected originated either from Mofela or Monong waterhole, where large groups of vultures were frequently observed during both sampling seasons. *Gyps coprotheres* DNA was detected in all sampling events where elephant DNA was present, but not in sampling events where nyala DNA was detected. Alternatively, the DNA could have been introduced to the reserve by visitor vehicles, potentially adhering to the tires after visits to those game parks. Secondary eDNA input into the study system as it could have occurred here has already been pointed out as an “overlooked source of false positives” for fishes in aquatic urban systems (Xiong et al. 2024), but has not yet been investigated thoroughly in terrestrial systems.

Additionally, three detected bird species are unlikely to occur in or around the game reserve, namely the mallard ( *Anas platyrhynchos* ), northern pintail ( *Anas acuta* ) and Hartlaubs' duck (*Pternonetta harlaubii*). None of these species has been observed during field work. However, two other *Anas* species, the yellow‐billed duck ( *Anas undulata* ) and red‐billed teal ( *Anas erythrorhyncha* ) were frequent visitors of the waterholes and are listed among the top 100 abundant birds for that area (African Bird Atlas Project 2022), both of which lack genetic reference sequences of both target fragments. We therefore hypothesize that the mallard and pintail detections arose from taxonomic misclassifications from one of these (or other) *Anas* species without genetic reference sequences. Missing genetic reference data is a persisting topic in eDNA metabarcoding studies, particularly for understudied taxa (Keck et al. 2023; Schenekar et al. 2020). Finally, Hartlaub's duck has only a single recorded occurrence in South Africa in the GBIF database, with its primary distribution range in Central and West Africa. This makes its presence in Botsalano Game Reserve highly unlikely. We attribute the detection of this species to a misclassification of the knob‐billed duck ( *Sarkidiornis melanotos* ), which does occur in the area of the reserve. The 12S rRNA target fragment of these two species differs by only one base pair, which resulted in a slightly better match with Hartlaub's duck (98/101 bp) than the knob‐billed duck (97/101 bp). Such low genetic resolution of metabarcoding markers is a frequent issue, particularly for young and/or sister species, and in such cases the utilization of other or additional metabarcoding markers has been recommended (Keck et al. 2023).

Regarding the detection of *L. mesomelas* , an abundant resident species in the reserve, we would like to note that although we are confident in our justification for the re‐assignment of the ASVs from *C. lupus* to this species (Material S3) and we consider secondary DNA input of *C. lupus* very unlikely, we cannot completely rule out this possibility and that a small part of the detections might indeed originate from domestic dog DNA, since we detected DNA of other domestic animals at very low frequencies, as well (0.14%–2.34% detection frequencies).

Concerning false negatives, despite conducting an extensive eDNA sampling campaign in the reserve, evidence suggests that not all resident species were detected. A concurrent camera trapping study (Sedlmayr et al. in prep) documented several low‐abundance mammal species absent in our eDNA data, such as caracal ( *Caracal caracal* ), serval ( *Leptailurus serval* ), and aardwolf ( *Proteles cristata* ), likely due to limited eDNA deposition in waterhole areas. Furthermore, while we detected all but one large mammal species (common reedbuck, *Redunca arundinum* ) from the Botsalano mammal species list (Morris 2022), that list excluded small mammals, which were not assessed in the reserve. Small mammals are particularly prone to false negatives due to their smaller body sizes, resulting in weaker eDNA signals, and the scarcity of their genetic reference sequences. Siziba and Willows‐Munro (2024) report that only 23.8% of South African small mammals have 12S rRNA reference sequences available, while our own gap analysis revealed approximately 50% coverage for South African mammals (Schenekar et al. 2024). While we are not aware of a gap analysis of birds, reptiles or amphibians for 12S rRNA sequences, we suspect the genetic reference sequences of these groups to be even less complete. Furthermore, the high proportion of ASV in our dataset assigned to species level or higher supports the risk of potential false negatives in our data due to incomplete reference databases. Additionally, none of the species accumulation curves flattened completely, indicating continued detections of new species with increasing sampling effort. While some rare detections might involve migratory birds, some of them originate from rare resident species potentially pointing to undetected resident species. Finally, most species detections originated from a single sample per sampling event (Figure S4), emphasizing low detection probabilities which could only be alleviated with substantially increased sampling effort.

### 4.2 Effect of Workflow Variations

#### 4.2.1 Sampled Substrate

Water and sediment samples showed similar overall species richness and taxonomic compositions, though with differences in detection patterns regarding species abundance. Sediment samples yielded higher detection frequencies for abundant species across sampling events, whereas water samples were more effective at detecting rare taxa (Figure 2). Furthermore, sediment samples exhibited higher species richness per sampling event (Figure 7a) and displayed a flatter decline of number of positive samples per species detection (Figure S4), indicating the more consistent detection of abundant taxa across samples and sampling events. In contrast, water samples yielded a greater overall species richness across the same number of sampling events (Figure 7b), showing a higher number of rare, exclusive species detections from water samples but also higher variability in species composition across sampling events. This pattern may result from the longer eDNA persistence in sediment (Corinaldesi et al. 2005; Sedlmayr and Schenekar 2024; Turner et al. 2015) and the higher concentration of abundant taxa eDNA along shorelines, which may outcompete rare taxa in metabarcoding. The larger water sample volumes (compared to sediment) may have counteracted eDNA patchiness and increased the detection of rare DNA fragments from low‐abundance species (Altermatt et al. 2023; Sepulveda et al. 2019). This is also consistent to observed trend from our water samples that larger filtered water volumes were associated with an increased number of detected species from these samples.

Soil samples collected from wildlife trails around waterholes yielded a lower species richness overall but were collected at a much lower sampling number than water and sediment samples. However, mammalian species richness was comparable to that detected from water and sediment samples. The reduced overall species richness primarily resulted from fewer bird detections. While terrestrial mammals frequently use these trails to access waterholes, depositing eDNA in the soil, bird eDNA is more abundant directly at the waterhole, where eDNA deposition results from direct interactions with the water, such as drinking or defecating while standing in water or being perched on overhanging branches. Thus, wildlife trails around waterholes may serve as viable alternative sampling sites for monitoring terrestrial mammals, particularly when direct sampling at waterholes is impractical due to inaccessibility or the presence of potentially dangerous animals (such as crocodile *Crocodylus niloticus* , hippopotamus— *Hippopotamus amphibius* or elephants— *Loxodonta africana* ) at the time of sampling.

#### 4.2.2 Seasonality

Overall, we detected more vertebrate species in the wet season than the dry season, but more mammals were detected during the dry season. While differences in filter types used for sampling between seasons cannot be ruled out as a contributing factor, we primarily attribute this pattern to two main ecological factors: (1) The increased biological activity of birds, amphibians and reptiles during the wet season, which coincides with the peak reproductive period of most species (Moreau 1950), led to higher detection probabilities for these taxa. (2) For non‐migratory mammals that also do not hibernate through the dry season, the reduced number of water bodies in the reserve (only two waterholes remained at the end of the dry sampling season) led to a higher utilization of the sampled waterholes, increasing detection probabilities and overall detected mammalian species richness.

#### 4.2.3 Primer Pair

The two primer pairs tested had the strongest influence on the detected species composition, with virtually non‐overlapping NMDS areas occupied (Figure 5d) and only 47% of species overlap (Figure 3). The vertebrate‐specific 12SV5 primer pair captured a broader range of vertebrate diversity, whereas the mammal‐specific MiMammal primer pair identified more mammal species. However, notably, the 12SV5 primer detected five mammal species that the MiMammal primer pair did not, while the MiMammal primer pair detected 31 non‐mammal vertebrates, including 10 undetected by 12SV5.

Metabarcoding primer choice is a critical consideration for any eDNA study, as multiple factors influence the success of species detection: (1) Taxonomic breadth and resolution: Selected primers should ideally target the specific taxonomic group of interest to avoid PCR bias, such as amplification efficiency differences affecting detection (Schenekar et al. 2020). Additionally, primers must provide adequate genetic resolution to distinguish closely related species. In our case, one primer pair was designed for mammals while the other targeted vertebrates. As expected, the vertebrate primers detected greater species diversity beyond mammals but also detected individual mammal species missed by the mammal‐specific primer, while the latter also detected non‐mammal vertebrates not picked up by the broader primer. This exemplifies the unpredictability in taxonomic coverage and resolution of primers. Methods such as in vitro tests (e.g., mock communities; Macher et al. 2023) or in silico analysis (e.g., sequence database screening; Schenekar et al. 2020) can aid in refining primer selection. (2) Amplicon length: Short DNA fragments are more abundant in environmental samples and therefore shorter amplicons may reduce false negatives (Bylemans et al. 2018). Both our primer pairs target short amplicons (< 200 bp). However, shorter fragments can reduce taxonomic resolution, potentially leading to misassignments or limits in identifying taxa to the species level, as observed in our dataset. (3) Gene region: Most metabarcoding primers target mitochondrial amplicons due to their high abundance in eDNA and presumed higher stability (Bylemans et al. 2018), though the specific gene region (e.g., 12S rRNA, COI, cytochrome b) affects genetic resolution (Clarke et al. 2017). Both primer pairs we employed targeted the 12S rRNA fragment, which is relatively conserved, reducing variability in amplification bias. Furthermore, the completeness of genetic reference databases differs across genes, with COI having by far the most complete database due to global Barcode of Life efforts (Hebert and Ratnasingham 2007). Despite COI's wide use in barcoding, it exhibits relatively high variability and has therefore been discouraged for metabarcoding applications (Deagle et al. 2014). While it is unclear whether combining primer pairs targeting different gene regions enhances detection, our results, originating from two primer pairs targeting 12S rRNA, are consistent with previous studies (McElroy et al. 2020; Wang et al. 2023) recommending the use of at least two primer pairs to maximize species detection in eDNA metabarcoding studies.

## 5 Conclusions and Recommendations

The findings of this study provide guidance for optimizing eDNA metabarcoding sampling designs around waterholes in semi‐arid or arid systems, like savannas, targeting terrestrial vertebrates. Regarding substrate choice, sediment samples performed superior in the consistent detection of abundant species, independent of taxonomic class (e.g., birds or mammals), while water samples were more effective for detecting rare species. Sediment sampling was quicker and required minimal equipment—gloves, plastic spatulas, and sampling tubes—with limited bleaching. In contrast, filtering a single water sample took up to 25 min, including pre‐filtering, filtration, and equipment handling. Sediment samples were processed in under 2 min by adding Longmire buffer, sealing, and shaking, making them significantly more field efficient. Therefore, for studies aiming to broadly assess species composition in a new or poorly studied system—or where field conditions limit sample processing logistics, such as remote locations or extensive daily sampling—we recommend sediment sampling of water bodies due to its simplicity and efficiency. Conversely, if detecting rare taxa is a priority and field conditions permit timely water filtration, water samples should be preferred. A combined sampling approach incorporating both substrates is likely to yield the highest species richness.

Soil samples from wildlife trails near waterholes offer a promising alternative for monitoring terrestrial mammals when direct access to waterholes is not possible. However, they are less effective for detecting other taxa, such as birds, amphibians, and reptiles, which rely more on direct interactions with water for eDNA deposition.

Sampling during the dry season may enhance terrestrial mammal detection, as these species tend to concentrate around the limited remaining water sources. In contrast, the detection of other taxa may decrease due to lower biological activity or migration. Additionally, seasonal changes in physicochemical properties—particularly suspended particle load—can significantly impact sampling efficiency, necessitating workflow adjustments, such as selecting appropriate filter pore sizes for water filtration. Using two primer pairs had the greatest positive impact on detected species richness. To maximize species detection within a given budget, we recommend prioritizing two independent genetic markers—or more where feasible—over, e.g., adding an extra sampling season.

## Funding

This work was supported by the Austrian Science Fund (10.55776/P35059).

## Conflicts of Interest

The authors declare no conflicts of interest.

## Supporting information


**Figure S1:** Average, maximum and minimum daily temperature, as well as average monthly rainfall (precipitation) of the study area in the time frame of the fieldwork. The sampling periods of the two seasons (“wet” and “dry”) are indicated by the two green bars. Precipitation data was received from Botsalano Game Reserve management, and temperature data from the National Centers for Environmental Information/National Oceanic and Atmospheric Administration (NCEI/NOAA).
**Table S1:** Larger‐bodied mammals recorded in Botsalano game reserve in October 2020. From (Morris, 2022). This count did not target small animals such as rodents or bats.
**Figure S2:** Water (a) and sediment (b) sampling at the waterholes.
**Figure S4:** Histograms showing the frequencies of the number of positive samples once a species has been detected in a sampling event. Per sampling event, eight samples were collected for sediment and water samples, whereas for soil samples, six sampling replicates were collected.
**Table S9:** Pairwise sequence similarities (in %, based on p‐distances) of cytochrome b between individuals of the eastern (E) and southern (S) population of *L. mesomelas* . (D) indicates domestic dog reference sequence as origin.
**Table S10:** Pairwise sequence similarities (in %, based on p‐distances) between ASVs that had the best taxonomic match to *C. lupus* and were re‐assigned to *L. mesomelas* .

**Table S2:** Inital taxonomic assignments of detected taxa using MIDORI database (“Initial taxon assignment”), the respective taxonomic level, class and whether the taxon has records in South Africa from gbif (“occuring in South Africa”). For taxa that could only be assigned to genus level and for species without gbif occurences in South Africa, the number of congeneric documented species in South Africa are given, as well as the number of congeneric documented species lacking genetic reference sequences (separately for “genus only” assignments and “non‐documentede species”). Furthermore, the number of congeneric species of the same genus known to occur in Botsalano Game Reserve are given for non‐documented large mammals. Based on this, it was noted whether the genus or the non‐documented species could be unambiguously assigned to one documented species and the final taxon name (“scientific name final” and “common name final”) are listed. Furthermore, the reason for the reassignment or the exclusion of the taxa from the dataset are given.

**Table S3:** Details on the collected samples. Given are the sample names, the type of sample, the substrate the samples were collected from, the sampling location, as well as the sampling spot within each location, the collection date, samplin season, filter type, whether the sample was prefiltered or not, and the proccessed volume (filtered water volume for water samples, collected sample volume for sediment and soil samples).

**Table S4:** Read counts assigned to ASVs for the individual samples. “Total read count” refers to reads that could be assigned to any of the filtered ASVs, whereas “species/genus” read count refers only to reads assigned to filtered ASVs that could be classified to the species or genus level.

**Table S5:** Mean read numbers with standard deviation (SD) for the individual sample types for both, the 12SV5 dataset and the MiMammal dataset.

**Table S6:** Number of species for the individual classes detected across different locations, substrate and seasons. Data is given for 12SV5 and MiMammal datasets, as well as the combined dataset.

**Table S7:** Absolute counts and relative frequencies of species detections across sampling events, for all sampling events combined (overall), and separated by substrates (sediment, soil and water). For each species, the scientific name (species), the common name and the family are given.

**Material S1.** Contains Figures S1–S4 and Table S1.
**Material S2.** Detailed extraction protocol.
**Material S3.** Details on ASV sequences re‐assigned from *C. lupus* to *L. mesomelas* .
**Material S4.** Bash and R codes used for bioinformatic and statistical analyses.

## Data Availability

The sequencing data of this study have been deposited in the GenBank Sequence Read Archive (SRA) under accession number PRJNA1218494 and can be accessed at https://www.ncbi.nlm.nih.gov/sra/PRJNA1218494. The bash codes for bioinformatic analysis, as well as R codes and input files for statistical analysis, are provided in Material S4. *Benefits generated*: A research collaboration was developed with scientists from Austria and South Africa, and all scientists actively contributing to logistic organization, data generation, data analysis, and manuscript writing are either listed as co‐authors or mentioned in the acknowledgements. The results of this collaborative project are being frequently shared with all collaborators and the broader scientific community. The research addresses a priority research focus of our South African collaborator (SANBI), namely biodiversity monitoring in South Africa. Sampling for the project has been approved by North West Parks Board and by the Department of Economic Development, Environment, Conservation and Tourism, South Africa (Permit No. NW 39881/05/2022), the research has been approved by SANBIs NZG Animal Research Ethics and Scientific Committee (P2022/15) and a permission in terms of section 20 of the animal diseases act 1984 was received from the Department of Agriculture, Land Reform and Rural Development, South Africa (DALRRD). A certificate of compliance in accordance with Article 17, paragraph 2, of the Nagoya Protocol has been issued by the Access and Benefit‐Sharing Clearing‐House (ABSCH) of the Department of Forestry, Fisheries and the Environment, South Africa (ABSCH‐IRCC‐ZA‐262932‐1).
