# Supplementary material for: Unveiling Vertebrate Biodiversity in Arid and Semi‐Arid Terrestrial Ecosystems Through eDNA Metabarcoding at Savanna Waterholes
Source: Evol Appl. 2026 Jan 29;19(2):e70200. doi: 10.1111/eva.70200 (PMC12855164; doi:10.1111/eva.70200)
Supplement: Supplementary file 8 — Material S1. Contains Figures S1–S4 and Table S1. Material S2. Detailed extraction protocol. Material S3. Details on ASV sequences re‐assigned from C. lupus to L. mesomelas . Material S4. Bash and R codes used for bioinformatic and statistical analyses. [file EVA-19-e70200-s004.zip › Supplementary material 2.docx]

**Supplementary material 2 -**

**Extraction protocol on eDNA filters in Longmire buffer using Qiagens DNeasy PowerSoil Pro Kit**

From:

Schenekar, T., Baxter, J., Andries, M., Sedlmayr, I., Weckworth, B., & Mwale, M. (2024). Optimizing waterborne eDNA capture from waterholes in savanna systems under remote field conditions. *Mol Ecol Res*, 1–19. https://doi.org/10.1111/1755-0998.13942

1. Spin down Power Bead Pro tube
2. Add 800 µl Buffer CD1 to Power Bead Pro tube
3. Remove filter from sample tube trying to take as little liquid as possible. Cut filter into 1-2 mm pieces using sterile forceps, scalpel and a weighing boat
4. Transfer cut filter into the Power Bead Pro tube
5. Homogenize sample at 4 rpm for 60 sec using a MP FastPrep 24 Homogenizer
6. Centrifuge at max speed for 1 min
7. Transfer supernatant into a 2 ml microcentrifuge tube
8. Add 200 µl Buffer CD2
9. Vortex for 5 sec
10. Centrifuge at max speed for 1 min
11. Transfer 700 µl of the supernatant into a new 2 ml microcentrifuge tube
12. Add 600 µl Buffer CD3
13. Vortex for 5 sec and spin down
14. Load 650 µl onto an MB spin column. Keep the remaining 650 µl, as well
15. Centrifuge MB spin column at max speed for 1 min
16. Empty collection tube and reinsert spin column
17. Repeat steps 14-16 one more time with the remaining 650 µl
18. Transfer spin column to a new 2 ml collection tube
19. Add 500 µl solution EA
20. Centrifuge at max speed for 1 min
21. Empty collection tube and reinsert spin column
22. Add 500 µl solution C5
23. Centrifuge at max speed for 1 min
24. Transfer spin column to a new 2 ml collection tube
25. Centrifuge at max speed for 2 min
26. Transfer spin column to a 1.5 ml elution tube
27. Add 100 µl solution C6 (heated to 56°C) directly onto the filter membrane
28. Incubate at room temperature for 1 min
29. Centrifuge at max speed for 1 min
30. Repeat steps 27-29 a second time (total elution volume: 200 µl)
