# Supplementary material for: Unveiling Vertebrate Biodiversity in Arid and Semi‐Arid Terrestrial Ecosystems Through eDNA Metabarcoding at Savanna Waterholes
Source: Evol Appl. 2026 Jan 29;19(2):e70200. doi: 10.1111/eva.70200 (PMC12855164; doi:10.1111/eva.70200)
Supplement: Supplementary file 8 — Material S1. Contains Figures S1–S4 and Table S1. Material S2. Detailed extraction protocol. Material S3. Details on ASV sequences re‐assigned from C. lupus to L. mesomelas . Material S4. Bash and R codes used for bioinformatic and statistical analyses. [file EVA-19-e70200-s004.zip › Supplementary material 3.docx]

**Supplementary material 3**

**Details on sequence similarity of ASVs generated by the two datasets of 12SV5 and MiMammal initially matching to *Canis lupus* (domestic dog/ grey wolf) that were re-assigned to *Lupulella mesomelas* (Black-backed jackal).**

At the date of publication, only one genetic reference sequence was available for the 12S rRNA fragment for *L. mesomelas* that covers the amplicons of the two primer pairs (12SV5 and MiMammal), (GenBank Accession number KT448280.1; Koepfli et al., 2015). This sequence originates from a sample of the “eastern” population of *L. mesomelas* (distributed in Eastern Africa such as Kenya and Tanzania), which is estimated to have split from the “southern” population of *L. mesomelas* (distributed in Southern Africa, such as South Africa, Namibia and Botswana) approximately 2.5 million years ago (Atickem et al., 2018). Those two populations show, on average, a sequence similarity of 92.31%, based on mitochondrial cytochrome b sequences (no information available for 12S sequences). Furthermore, the similarity of the southern clade to the domestic dog reference was marginally higher (90.5%) compared to the eastern clade (89.7%, Table S9).

**Table S9** Pairwise sequence similarities (in %, based on p-distances) of cytochrome b between individuals of the eastern (E) and southern (S) population of *L. mesomelas*. (D) indicates domestic dog reference sequence as origin

| GenBank Access. # (origin) | KT447688 E | MF196943 E | MF196944 E | MF196945 E | MF196946 E | MF196947 E | MF196948 E | MF196959 E | MF196950 E | MF196951 S | MF196953 S | MF196954 S | MF196955 S | MF196956 S | MF196957 S | MF196958 S |
| --- | --- | --- | --- | --- | --- | --- | --- | --- | --- | --- | --- | --- | --- | --- | --- | --- |
| KT447688 E | - |  |  |  |  |  |  |  |  |  |  |  |  |  |  |  |
| MF196943 E | 100 | - |  |  |  |  |  |  |  |  |  |  |  |  |  |  |
| MF196944 E | 100 | 100 | - |  |  |  |  |  |  |  |  |  |  |  |  |  |
| MF196945 E | 99.90 | 99.90 | 99.90 | - |  |  |  |  |  |  |  |  |  |  |  |  |
| MF196946 E | 99.90 | 99.90 | 99.90 | 99.79 | - |  |  |  |  |  |  |  |  |  |  |  |
| MF196947 E | 99.90 | 99.90 | 99.90 | 99.79 | 100 | - |  |  |  |  |  |  |  |  |  |  |
| MF196948 E | 99.58 | 99.58 | 99.58 | 99.48 | 99.48 | 99.48 | - |  |  |  |  |  |  |  |  |  |
| MF196959 E | 99.48 | 99.48 | 99.48 | 99.38 | 99.38 | 99.38 | 99.90 | - |  |  |  |  |  |  |  |  |
| MF196950 E | 99.48 | 99.48 | 99.48 | 99.38 | 99.38 | 99.38 | 99.90 | 100 | - |  |  |  |  |  |  |  |
| MF196951 S | 92.62 | 92.62 | 92.62 | 92.52 | 92.52 | 92.52 | 92.41 | 92.31 | 92.31 | - |  |  |  |  |  |  |
| MF196953 S | 92.41 | 92.41 | 92.41 | 92.31 | 92.31 | 92.31 | 92.20 | 92.10 | 92.10 | 99.17 | - |  |  |  |  |  |
| MF196954 S | 92.41 | 92.41 | 92.41 | 92.31 | 92.31 | 92.31 | 92.20 | 92.10 | 92.10 | 99.17 | 100 | - |  |  |  |  |
| MF196955 S | 92.41 | 92.41 | 92.41 | 92.31 | 92.31 | 92.31 | 92.20 | 92.10 | 92.10 | 99.17 | 100 | 100 | - |  |  |  |
| MF196956 S | 92.52 | 92.52 | 92.52 | 92.41 | 92.41 | 92.41 | 92.31 | 92.20 | 92.20 | 99.69 | 99.48 | 99.48 | 99.48 | - |  |  |
| MF196957 S | 92.52 | 92.52 | 92.52 | 92.41 | 92.41 | 92.41 | 92.31 | 92.20 | 92.20 | 99.69 | 99.48 | 99.48 | 99.48 | 100 | - |  |
| MF196958 S | 92.20 | 92.20 | 92.20 | 92.10 | 92.10 | 92.10 | 92.00 | 91.89 | 91.89 | 99.38 | 99.17 | 99.17 | 99.17 | 99.69 | 99.69 | - |
| MW5649038 D | 89.71 | 89.71 | 89.71 | 89.60 | 89.81 | 89.81 | 89.71 | 89.60 | 89.60 | 90.85 | 90.44 | 90.44 | 90.44 | 90.54 | 90.54 | 90.23 |

From our metabarcoding data, six ASVs were assigned to *C. lupus* from both, the MiMammal dataset (ASVs 188, 35155 and 44277) and the 12SV5 dataset (ASVs 1535, 75246 and 2187) while one ASV was assigned to *L. mesomelas* (ASV7643).

These six ASVs had a best taxonomic match to *C. lupus*, with an average of 96.2% sequence identity to *C. lupus* and 93.06% sequence identity to the one *L. mesomelas* reference sequence (of the “eastern” population of *L. mesomelas*, Table S10). Notably, also ASV 7643, that had the best match with *L. mesomelas*, shows a sequence similarity of only 93.45% to *L. mesomelas* (and 91.67% with *C. lupus*), as well.

**Table S10** Pairwise sequence similarities (in %, based on p-distances) between ASVs that had the best taxonomic match to *C. lupus* and were re-assigned to *L. mesomelas*

|  | *C. lupus* MW549038.1 | *L. mesomelas* KT448280.1 | MiMammal ASV7643 (assigned to *L. mesomelas*) | MiMammal ASV188 (assigned to *C. lupus*) | MiMammal ASV35155 (assigned to *C. lupus*) | MiMammal ASV44277 (assigned to *C. lupus*) | 12SV5 ASV1535 (assigned to *C. lupus*) | 12SV5 ASV75246 (assigned to *C. lupus*) | 12SV5 ASV2187 (assigned to *C. lupus*) |
| --- | --- | --- | --- | --- | --- | --- | --- | --- | --- |
| *C. lupus* MW549038.1 | - |  |  |  |  |  |  |  |  |
| *L. mesomelas* KT448280.1 | 96.44 | - |  |  |  |  |  |  |  |
| MiMammal ASV7643 (assigned to *L. mesomelas*) | 91.67 | 93.45 | - |  |  |  |  |  |  |
| MiMammal ASV188 (assigned to *C. lupus*) | 100.00 | 95.86 | 91.67 | - |  |  |  |  |  |
| MiMammal ASV35155 (assigned to *C. lupus*) | 97.02 | 93.45 | 91.02 | 97.02 | - |  |  |  |  |
| MiMammal ASV44277 (assigned to *C. lupus*) | 96.45 | 92.31 | 88.10 | 96.45 | 93.45 | - |  |  |  |
| 12SV5 ASV1535 (assigned to *C. lupus*) | 92.93 | 90.91 | n/a | n/a | n/a | n/a | - |  |  |
| 12SV5 ASV75246 (assigned to *C. lupus*) | 91.92 | 90.91 | n/a | n/a | n/a | n/a | 84.85 | - |  |
| 12SV5 ASV2187 (assigned to *C. lupus*) | 98.99 | 94.95 | n/a | n/a | n/a | n/a | 91.92 | 92.93 | - |

Therefore, even though the sequences of these ASVs have a higher sequence identity with *C. lupus* that causes the initial taxonomic assignment to this species, they fall well within the documented mitochondrial sequence differences between the eastern population (origin of the available reference sequence) and the southern population (geographically occurring in our study area).

Additionally, *C. mesomelas* has a documented abundance in the reserve (Morris, 2022) and we received frequent observations on images of a concurrent camera trapping study (Sedlmayr et al., in prep), matching our own personal observation of the widespread presence of this species in the reserve during field work. Prohibition of dogs in the reserve paired with the high detection frequency of the taxon originating from these ASVs (in 57% of sampling events, Fig. 2d, detection frequency across field samples 15.45%), whereby detection from other domestic animals fell orders of magnitude below this frequency, (0.14-2.34%) lets us conclude that the initially assignment of these ASVs to *C. lupus* originates from a taxonomic misassignment due to missing reference sequences of the southern population of *C. lupulella*, emphasising the need of multiple genetic references to account for deep genetic divergence between populations and across the distribution area of a species.

References

Atickem, A., Stenseth, N. C., Drouilly, M., Bock, S., Roos, C., & Zinner, D. (2018). Deep divergence among mitochondrial lineages in African jackals. *Zoologica Scripta*, *47*(1), 1–8. https://doi.org/10.1111/zsc.12257

Koepfli, K. P., Pollinger, J., Godinho, R., Robinson, J., Lea, A., Hendricks, S., Schweizer, R. M., Thalmann, O., Silva, P., Fan, Z., Yurchenko, A. A., Dobrynin, P., Makunin, A., Cahill, J. A., Shapiro, B., Álvares, F., Brito, J. C., Geffen, E., Leonard, J. A., … Wayne, R. K. (2015). Genome-wide evidence reveals that African and Eurasian golden jackals are distinct species. *Current Biology*, *25*(16), 2158–2165. https://doi.org/10.1016/j.cub.2015.06.060

Morris, J. W. (2022). *Botsalano Game Reserve - A Visitor Guide* (p. 36).

Sedlmayr, I., Baxter, J., Letlojane, I., Mwale, M., Schenekar, T. *Monitoring mammals at waterholes in the savanna - a comparison between eDNA and camera trapping* (in prep).
